# Calpain-Mediated Cleavage of Calcineurin in Puromycin Aminonucleoside-Induced Podocyte Injury

**DOI:** 10.1371/journal.pone.0155504

**Published:** 2016-05-12

**Authors:** Fangrui Ding, Xuejuan Li, Baihong Li, Jifan Guo, Yanqin Zhang, Jie Ding

**Affiliations:** Department of Pediatrics, Peking University First Hospital, Beijing, China; Sungkyunkwan University, REPUBLIC OF KOREA

## Abstract

The calcineurin inhibitors cyclosporine A (CsA) and tacrolimus are widely used in the treatment of proteinuria diseases. As the direct target of these drugs, calcineurin has previously been demonstrated to play a role in proteinuria diseases. However, aside from its immune-related effects, the local status of calcineurin in renal inherent cells has not been fully explored in the settings of proteinuria disease and podocyte injury. In this study, calcineurin activity and protein expression in the well-known puromycin aminonucleoside (PAN)-induced podocyte injury model were examined. Interestingly, we found that calcineurin activity was abnormally increased in PAN-treated podocytes, whereas the expression of the full-length 60-kDa calcineurin protein was decreased. This result suggests that there may be another activated form of calcineurin that is independent of the full-length phosphatase. To investigate whether calpain is involved in regulating calcineurin, we exposed PAN-treated podocytes to both pharmacological inhibitors of calpain and specific siRNAs against calpain. Calpain blockade reduced the enhanced calcineurin activity and restored the down-regulated expression of 60-kDa calcineurin. In addition, purified calpain protein was incubated with podocyte extracts, and a 45-kDa fragment of calcineurin was identified; this finding was confirmed in PAN-induced podocyte injury and calpain inhibition experiments. We conclude that calcineurin activity is abnormally increased during PAN-induced podocyte injury, whereas the expression of the full-length 60-kDa calcineurin protein is down-regulated due to over-activated calpain that cleaves calcineurin to form a 45-kDa fragment.

## Introduction

The immunosuppressive drugs cyclosporine A (CsA) and tacrolimus are widely used in renal proteinuria diseases, including minimal change disease (MCD), to reduce excessive protein levels in urine [1–4]. The initial motivation for using these drugs was based on the understanding that MCD pathogenesis involves dysregulated production of several circulating immune factors by T cells, which are eventually deposited in glomeruli [5]. However, many studies have shown that abnormalities in glomerular inherent cells, including podocytes, play key roles in proteinuria diseases independent of immune factors [6, 7]. Genetic and functional studies have revealed that mutation of a single podocyte-specific gene, such as NPHS1 or NPHS2, is sufficient to cause proteinuria [8, 9]. Moreover, both human and experimental studies have shown that CsA can reduce proteinuria in some nonimmunological diseases, including Alport syndrome [10, 11]. Collectively, these data suggest that immunosuppressive drugs have a direct effect on the kidney beyond that of immunoregulation. In 2008, Faul *et al*. performed an elegant study demonstrating that CsA has a direct impact on the actin cytoskeleton of podocytes and has anti-proteinuria effects. CsA, an inhibitor of calcineurin, can block calcineurin-mediated dephosphorylation of synaptopodin, which then results in the stabilization of the actin cytoskeleton in podocytes and the reduction of proteinuria in renal diseases [12]. These findings suggest that the local status of calcineurin in glomeruli and podocytes should be considered in future studies on CsA and tacrolimus.

Calcineurin is a serine/threonine phosphatase composed of two subunits, a 60-kDa catalytic subunit termed calcineurin A (CnA), and a 19-kDa regulatory subunit termed calcineurin B (CnB). CnA has an autoinhibitory domain and a calmodulin-binding domain at the C-terminus and a catalytic domain and a CnB-binding domain at the N-terminus [13]. The abnormal activation and expression of calcineurin are involved in many nervous system and cardiovascular system diseases [14, 15]. The well-characterized activation of calcineurin occurs after calcium and calmodulin bind to a site in CnA. These binding events trigger the release of the autoinhibitory domain from the catalytic active site, resulting in calcineurin activation, which occurs in a calmodulin-dependent manner [16]. Nevertheless, in several neurodegenerative diseases, calcineurin can also be activated by proteolytic cleavage of the autoinhibitory domain. Because the autoinhibitory domain is removed by proteolysis and the catalytic domain is exposed, the phosphatase activity of calcineurin is then activated in a calmodulin-independent manner [17]. Calpain is involved in cleaving the 60-kDa full-length CnA and converting it into active fragments in some nervous system diseases [17, 18]. Calpain, a calcium-activated protease, is constitutively activated and participates in the processes of glomerular injury and proteinuria [19]. The abnormality of calpain and calcineurin were separately involved in glomerular diseases. Thus, the local status of calcineurin and relationship between clacineurin and calpain in podocytes injury will be examined in present study.

In this study, we constructed a classical puromycin aminonucleoside (PAN)-induced podocyte injury model to further investigate the relationship between calpain and calcineurin during podocyte injury. We then applied both pharmacological inhibitors of and specific siRNAs targeted against calpain to determine whether calpain is involved in regulating calcineurin. Purified human calpain was also used to confirm its role in calcineurin cleavage.

## Results

### PAN-induced podocyte injury

**Fig 1:**
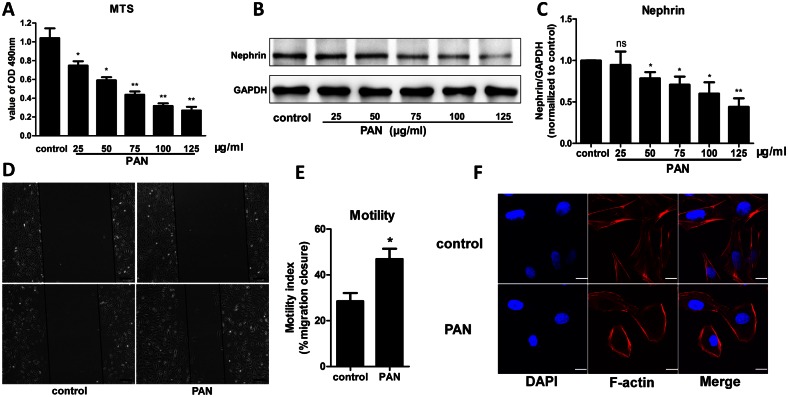
PAN-induced podocyte injury model. (A) Podocytes treated with PAN (25, 50, 75, 100, or 125 μg/ml) for 24 h showed a dose-dependent decrease in viability. (B, C) The protein expression of nephrin, a marker of podocyte injury, was decreased following PAN treatment at concentrations ≥50 μg/ml, demonstrating that a PAN concentration ≥50 μg/ml was sufficient to induce podocyte injury. (D, E) Podocytes were examined by microscopy at 0 and 24 h. After 24 h, podocytes that were treated with 75 μg/ml PAN migrated faster than the controls, suggesting that the motility of PAN-treated podocytes was abnormal. (F) Phalloidin and DAPI staining of untreated control podocytes showed fine F-actin stress fibers, whereas podocytes exposed to 75 μg/ml PAN show a loss of stress fibers and the appearance of a disordered and thick cortical stress fiber distribution. (*p<0.05 vs. control; **p<0.01 vs. control; ns, no statistical significance; n = 3. Black bar = 200 μm; White bar = 40 μm. PAN: Puromycin aminonucleoside)

The PAN model was constructed to study the status of calcineurin during podocyte injury. This classical model is used to study proteinuria diseases both *in vivo* and *in vitro* [20, 21]. First, an MTS assay was performed to observe the effect of PAN on podocyte viability. As shown in Fig 1A, the OD 490 nm value was decreased by PAN treatment in a dose-dependent manner, suggesting that PAN negatively affects podocyte viability. Additionally, in PAN-treated podocytes, there was a significant dose-dependent decrease in the protein expression of nephrin, a marker of podocyte injury (Fig 1B and 1C). Limited physiological motility and the presence of stress fibers are characteristics of normal podocytes. Enhanced motility and a loss of stress fibers are considered markers of injured podocytes[22–24]. The motility and stress fiber status of podocytes were evaluated using a migration assay and F-actin staining. As shown in Fig 1D–1F, PAN treatment significantly promoted podocyte migration and damaged stress fibers. These results suggest that a podocyte injury model was successfully generated with the application of PAN.

### Involvement of both calcineurin and calpain in podocyte injury

**Fig 2:**
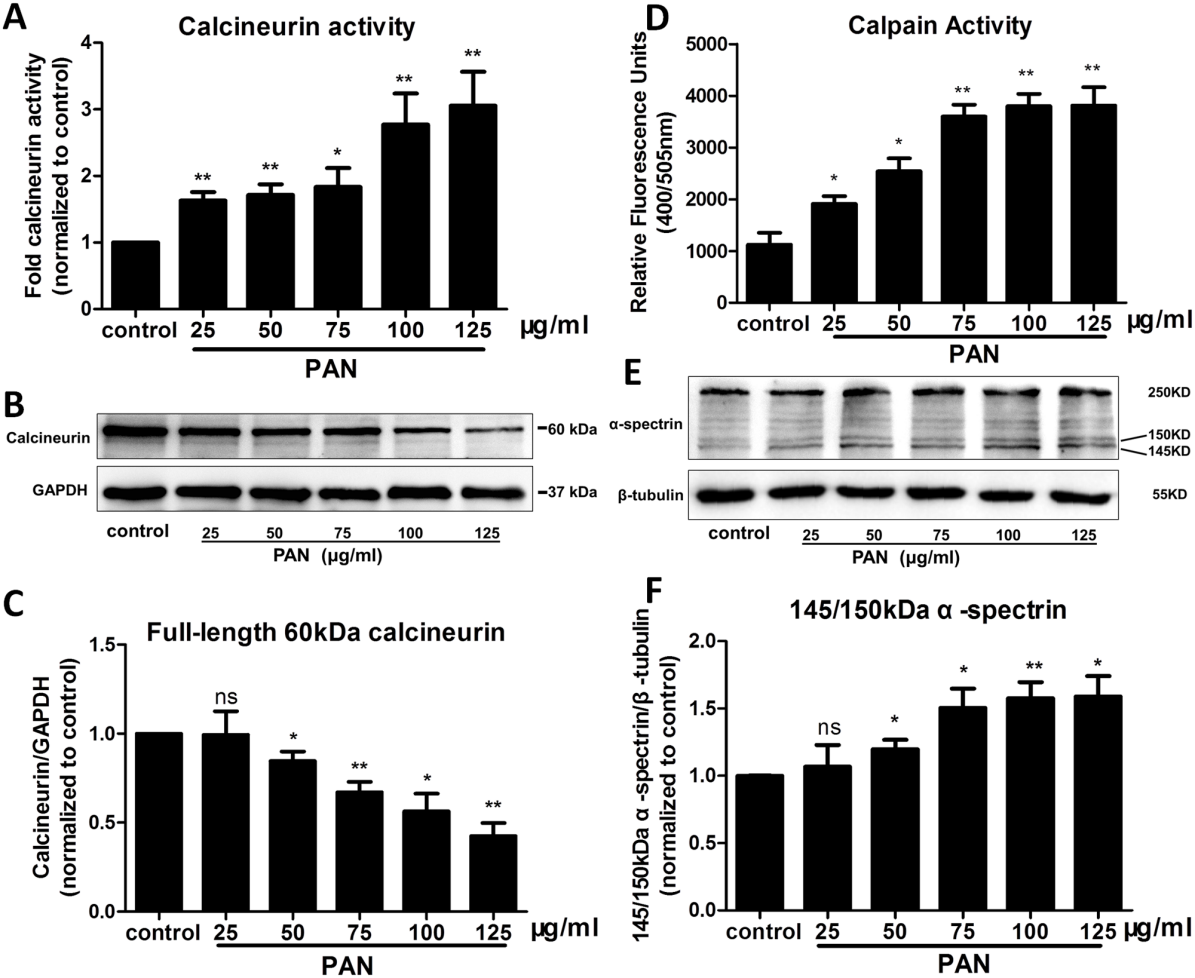
Calcineurin and calpain were abnormal in PAN-treated podocytes. (A) Enzyme activity of calcineurin in podocytes. Compared to untreated control podocytes, calcineurin activity was up-regulated in podocytes treated with various concentrations of PAN (25, 50, 75, 100, or 125 μg/ml) after 24 h. (B, C) Interestingly, the protein expression of full-length 60-kDa calcineurin was decreased in a dose-dependent manner in PAN-treated podocytes; this effect was the opposite of that observed on calcineurin activity. (D) Increased calpain activity was observed in PAN-treated podocytes. (E, F) Dose-dependent changes in the cleavage of non-erythroid α-spectrin after PAN exposure. The calpain-dependent truncation of α-spectrin from 250-kDa to 145-kDa and 150-kDa fragments suggests that calpain was activated. Increases in the abundance of the two smaller fragments indicated that calpain was abnormally activated in PAN-treated podocytes. (*p<0.05 vs. control; **p<0.01 vs. control; ns, no statistical significance; n = 3. PAN: Puromycin aminonucleoside)

Both calcineurin and calpain are enzymes that play important roles in physiological processes. To explore whether calcineurin and calpain are involved in podocyte injury, we evaluated the protein expression and activation of these molecules in podocytes. As shown in Fig 2A–2C, PAN increased calcineurin activity in a dose-dependent manner. Interestingly, the level of full-length 60-kDa calcineurin protein was decreased after treatment with various concentrations of PAN. This result suggests that there may be another activated form of calcineurin that is independent of the full-length phosphatase. Previous studies in the nervous system showed that calpain is involved in calcineurin cleavage, which results in the formation of another activated fragment [17]. Thus, calpain activity was also evaluated in the present study. Both a direct activity assay and indirect substrate detection were performed, and the results are shown in Fig 2D–2F. Upon treatment with PAN, calpain activity was increased compared with that observed in the control. In addition, to further confirm whether calpain activity was actually activated after PAN exposure, the expression of cleaved non-erythroid α-spectrin, a well-known substrate of calpain, was examined [25]. Upon PAN treatment, the full-length 250-kDa protein was cleaved into the characteristic 145-kDa and 150-kDa fragments in a dose-dependent manner.

### Calpain-mediated regulation of calcineurin during podocyte injury

**Fig 3:**
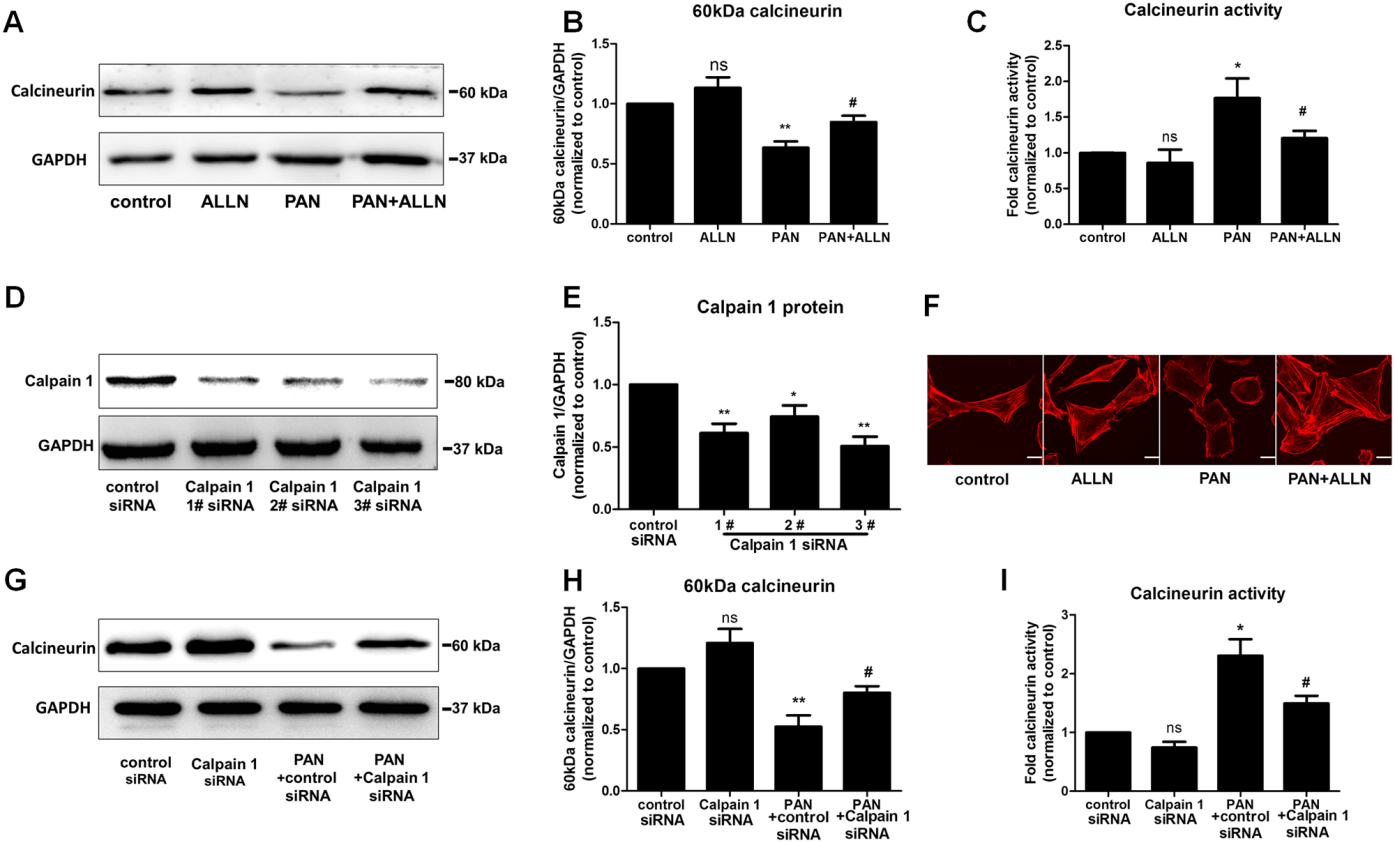
Calpain regulated both the expression and activity of calcineurin. (A, B, G, H) After PAN treatment, the level of full-length 60 KDa calcineurin in podocytes was significantly decreased (lane 3). Calpain inhibition by ALLN or calpain knockdown restored the normal level of full-length calcineurin in PAN-treated podocytes (lane 4). (C, I) Calcineurin activity was abnormally enhanced after PAN exposure, whereas calpain inhibition with ALLN pre-treatment and calpain knockdown in PAN-treated podocytes suppressed the enhanced activity of calcineurin. (D, E) Screening of highly effective calpain siRNAs. Calpain protein expression decreased significantly after transfection with calpain siRNAs, especially siRNA #3, compared to control siRNA. In our study, calpain knockdown was performed using siRNA #3, as shown in 3G, 3H and 3I. (F) Phalloidin staining of untreated control and ALLN treated podocytes showed fine F-actin stress fibers, whereas podocytes exposed to 75 μg/ml PAN show a loss of stress fibers and the appearance of a disordered and thick cortical stress fiber distribution. After bloackade of calpain by using ALLN in PAN treated podocytes, the F-actin was present and recovered to fine but still a little disordered and thick cortical stress fiber distribution. (*p<0.05 vs. control or control siRNA; **p<0.01 vs. control; #p<0.05 vs. PAN or PAN+control siRNA; ns, no statistical significance; n = 3. PAN: Puromycin aminonucleoside)

Both calcineurin and calpain were activated during podocyte injury. To investigate whether calpain is involved in the regulation of calcineurin during podocyte injury, we used pharmacological inhibitors of calpain and specific siRNAs targeted against calpain. After blockade of calpain with the calpain inhibitor ALLN in PAN-treated podocytes, the increased calcineurin activity was reduced, and the reduced calcineurin protein expression was restored (Fig 3A–3C). In addition, we used siRNAs specific for calpain 1 to further confirm the role of calpain in calcineurin regulation. To exclude off-target effects, three siRNAs against calpain 1 were used in our study. siRNA #3 showed the greatest calpain 1 knockdown and was chosen for subsequent experiments (Fig 3D and 3E). After silencing calpain 1, podocytes were treated with PAN for 24 h. An activity assay was performed, and protein expression was detected. As shown in Fig 3G–3I, calcineurin activity was increased after PAN exposure, and pre-treatment with a calpain 1-specific siRNA decreased calcineurin activity in podocytes compared to the PAN-only treatment group. In addition, the protein expression of full-length calcineurin was restored in PAN-treated podocytes with calpain 1 knockdown. In addition, we have stained the F-actin to confirm whether blockade of calpain had an effect on recovering injured podocytes. As shown in Fig 3F, the damaged stress fibers were recovered after calpain inhibition. Collectively, we concluded that calpain induced the decreasing of the full length 60KDa calcineurin during podocytes injury and blockade of calpain had an effect on recovering the injured podocytes.

### Identification of calpain-cleaved calcineurin during podocyte injury

**Fig 4:**
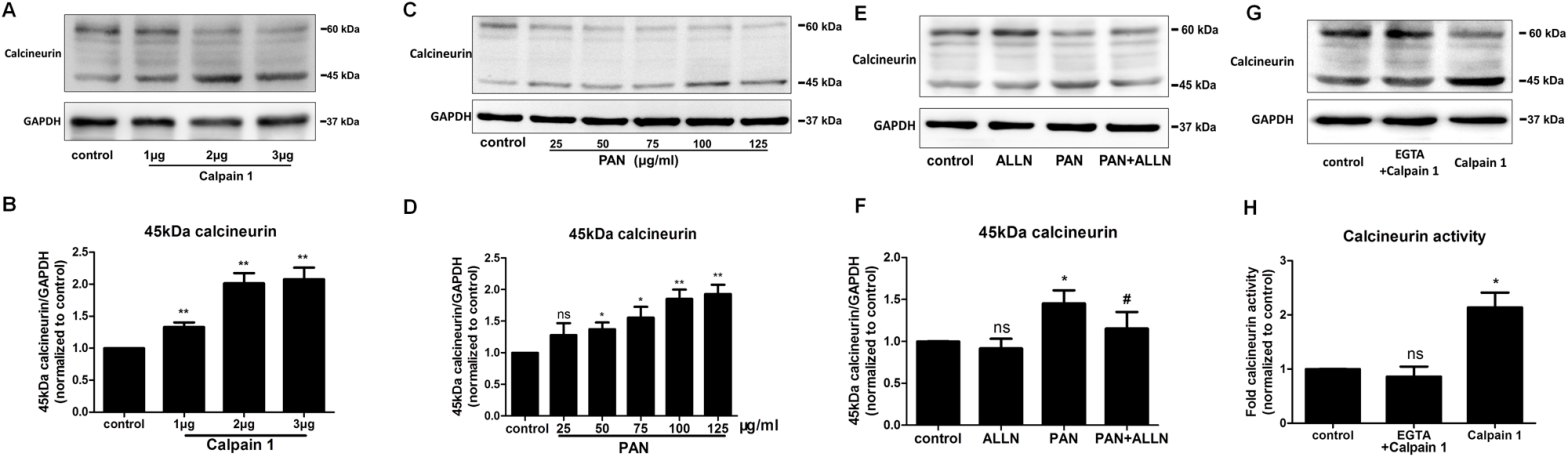
Calpain mediated the cleavage of calcineurin in PAN-treated podocytes. (A, B) The cleavage pattern of calcineurin was examined in the presence of purified calpain 1 *in vitro*. Purified calpain 1 (1, 2, or 3 μg) was incubated with 1 mM Ca^2+^ at 37°C for 30 min. After the incubation, each sample was loaded onto a 10% SDS-PAGE gel, and the proteins were examined by western blotting. A 45-kDa fragment was detected, and the expression of this fragment was increased at the expense of 60-kDa calcineurin. (C, D) The expression of cleaved 45-kDa calcineurin was dependent on the increased concentration of PAN (25, 50, 75, 100, and 125 μg/ml) in podocytes and was associated with a dose-dependent decrease in 60-kDa calcineurin. (E, F) Inhibiting calpain with ALLN resulted in a reduced level of the 45-kDa calcineurin cleavage product (lane 4), whereas enhanced calcineurin cleavage (lane 3) was observed in the podocytes treated with PAN only. (G, H) Purified calpain 1 (2μg) was incubated with podocytes extracts in presence of 2 mM EGTA to chelate all the free calcium at 37°C for 30 min. Also, purified calpain 1 was also incubated with podocytes extracts at the same time. After incubation, the western blotting showed that the single calpain without calcium (lane 2) cannot increase the cleavage 45KDa calcineurin and calcineurin activity. (*p<0.05 vs. control or control siRNA; **p<0.01 vs. control; #p<0.05 vs. PAN; ns, no statistical significance; n = 3. PAN: Puromycin aminonucleoside)

Blockade of calpain had an effect on recovering the full-length calcineurin. To confirm whether a cleaved fragment of calcineurin mediated by calpain is produced concomitant with the reduction in full-length calcineurin expression, Western blotting was performed to detect calcineurin fragments. First, purified human full-length calpain 1 protein was incubated with podocyte protein extracts. After incubating these extracts with calpain 1 in the presence of calcium, a 45-kDa cleavage product was detected, similar to that reported in previous studies on the nervous system [17]. As shown in Fig 4A and 4B, the full-length 60-kDa calcineurin protein was decreased in a dose-dependent manner by incubation with calpain 1, while the 45-kDa fragment showed a corresponding increase. Given this result, the full-length and cleaved forms of calcineurin were evaluated in PAN-treated podocytes to determine whether the 60-kDa calcineurin protein was cleaved into the 45-kDa fragment. As shown in Fig 4C and 4D, the expression of the 60-kDa full-length calcineurin was decreased, while that of the 45-kDa fragment was increased in a dose-dependent manner. Moreover, when PAN-treated podocytes were exposed to the calpain inhibitor ALLN, the level of the 45-kDa fragment was decreased compared with that observed in the PAN-only treatment group, and expression of the 60-kDa protein was restored. In addition, the activation of calpain was caused by calcium which also had a dependent effect on calcineurin with calmodulin, to examine whether the cleavage of calcineurin was caused by activated calpain independent of calcium, the EGTA which could chelate calcium was used to rule out the effect of calcium. As shown in Fig 4G, after chelating calcium with EGTA, the single calpain 1 had no effect on cleavage of calcineurin while without EGTA, cleaved calcineurin was increased. Since the calcineurin activity was also dependent on calcium, the activity of calcineurin was also examined with chelating calcium with EGTA, as shown in Fig 4H, in presence of EGTA, the activation of calcineurin by calpain was no difference with control. Hence, these results suggest that the calcineurin proteolysis and activation in the podocytes extracts resulted from activation of calpain 1.

## Materials and Methods

### Cell culture and drug treatment

The mouse podocyte cell line MPC5 used in our study was first established by Professor Peter Mundel et al. [26] in 1997, and it is widely used in many podocyte studies [26, 27]. The culture method for MPC5 podocytes was similar to the originally described method. Briefly, podocyte proliferation occurred in a 33°C incubator using RPMI 1640 proliferation medium (Gibco, USA) containing 10% fetal bovine serum (Gibco, USA), 100 U/ml penicillin-streptomycin (Gibco, USA) and 10 U/ml recombinant mouse interferon-γ (Sigma, USA). When the podocytes reached 60%-80% confluence, they were transferred to a 37°C incubator for differentiation for 10–14 days with RPMI 1640 medium in the absence of interferon-γ. The calpain inhibitor N-acetyl-Leu-Leu-Nle-CHO (ALLN) (Santa Cruz Biotechnology, USA) was dissolved in dimethyl sulfoxide (DMSO) (GeneChem, China), and the appropriate amount of DMSO was added to each control sample. PAN (Sigma, USA) was dissolved in water. Podocytes were treated with different concentrations of PAN (25, 50, 75, 100, or 125 μg/ml) for 24 h, and 75 μg/ml was selected as the appropriate concentration for subsequent studies, including migration assays, F-actin staining, and calpain inhibition experiments.

### Western blot

Total protein extracted from podocytes was lysed in radioimmunoprecipitation assay (RIPA) lysis buffer (50 mM Tris-HCl (pH 7.4), 150 mM NaCl, 1% Nonidet P-40, 0.1% sodium dodecyl sulfate (SDS), and 0.5% deoxycholic acid sodium salt (DOC)). Equal amounts of protein were subjected to 8–12% SDS-PAGE and electrophoretically transferred onto nitrocellulose membranes (Amersham Life Science, USA). After blocking with 5% non-fat milk in PBS containing 0.05% Tween-20 (PBST) for 1 h at room temperature, the membranes were incubated overnight at 4°C with one of the following primary antibodies: rabbit anti-nephrin (Sigma, USA), rabbit anti-calcineurin (Abcam, USA), rabbit anti-α-spectrin (Abcam, USA), rabbit anti-calpain 1 (Abcam USA), mouse anti-β-tubulin (Santa Cruz Biotechnology, USA), or mouse anti-β-actin (Santa Cruz Biotechnology, USA). The membranes were then rinsed 3 times for 8 min each in PBST and incubated with anti-rabbit or anti-mouse IgG secondary antibodies (Santa Cruz Biotechnology, USA). After a final washing step, the membranes were developed using an enhanced chemiluminescence reagent (Millipore, USA), and protein bands were scanned. Finally, images were collected and analyzed using ImageJ software (National Institutes of Health, USA).

### Cell viability assay

Podocytes were cultured in medium in 96-well plates and treated with or without PAN for 24 h. According to the manufacturer’s recommendations, 38 μg/ml MTS assay buffer was added to each well, and the absorbance at 490 nm was then read using a microplate reader (BioTek, USA). The absorbance values at 490 nm were exported to Excel and analyzed.

### Cell migration assays

Podocytes were plated on 6-well plates coated with type I collagen (Santa Cruz Biotechnology, USA). Each well was then scratched with a sterile 200-μl pipette tip, washed with PBS, and replenished with fresh medium. Images were captured at 0 h using an Olympus inverted microscope connected to a DP72 digital camera (Olympus, Japan). The cells were then immediately treated with or without 75 μg/ml PAN, and images were collected again after 24 h. The images were analyzed using ImageJ software to quantify cell migration (expressed as the percentage of migration area covered).

### Fluorescence confocal microscopy

Podocytes were plated on a confocal dish (NEST, UK) and treated with relevant treatment for 24 h. After treatment, the podocytes were washed three times with PBS and fixed with 4% paraformaldehyde, followed by permeabilization and blocking with 0.3% Triton X-100 and 10% goat serum. Alexa-phalloidin (Invitrogen, USA) was used to stain F-actin. After three washes with PBS, the nuclei were stained with 4',6-diamidino-2-phenylindole (DAPI) (Invitrogen, USA). Stained images were obtained by confocal laser-scanning microscopy (Zeiss, Germany).

### Calcineurin activity assay

Calcineurin activity was examined using a Cellular Calcineurin Phosphatase Activity Assay Kit (Colorimetric) (Abcam, USA). Briefly, podocytes were harvested into lysis buffer with a protease inhibitor provided in the assay kit. The samples were centrifuged at 120,000 × g in a high-speed centrifuge (Hitachi, Japan) at 4°C for 45 min to sediment the cells and remove the nuclei. The resulting supernatant was collected and then desalted using Desalting Resin provided in the assay kit to remove excess phosphate and nucleotides. After preparing the reaction buffer by adding 25 μl of 2X calcineurin assay buffer and 10 μl of calcineurin substrate to 96-well plates, 5 μl of each extract was added to the appropriate wells, and the plates were incubated at 30°C for 30 min. After incubation for the desired duration, 100 μl of Green Assay Reagent was added to each well to terminate the reactions. The plates were placed at room temperature for 20–30 min for color development, values were read at 620 nm on a microplate reader (BioTek, USA), and the data were subsequently analyzed.

### Calpain activity assay

Calpain activity in podocytes was examined using a Calpain Activity Assay Kit (Biovision, Germany) according to the manufacturer’s protocol. Briefly, podocytes were treated as described above and harvested by centrifugation. The podocytes were then resuspended in 100 μl of Extraction Buffer provided in the assay kit. The samples were incubated on ice for 20 min with gentle mixing by tapping several times during the incubation. Subsequently, the samples were centrifuged for 1 min at 10,000 × g, and the supernatant was transferred to a fresh tube. Protein concentrations were assayed using a microplate reader (BioTek, USA), and an equal amount of supernatant diluted in 85 μl of extraction buffer, 10 μl of 10X reaction buffer and 5 μl of calpain substrate provided in the assay kit were added to each assay. After an incubation at 37°C for 1 h in the dark, the samples were read in a fluorometer equipped with a 400-nm excitation filter and a 505-nm emission filter (BioTek, USA).

### Proteolysis of calcineurin by purified calpain

Podocyte extracts were prepared in HEPES homogenization buffer (pH 7.5) (Gibco, USA) with Okadaic Acid, the inhibitor of PP1 and PP2A phosphatases. The extracts were incubated in the presence of Calcium (1 mM) and purified human full-length calpain 1 protein (Abcam, USA) for 30 min at 37°C. The reactions were terminated by the addition of 5x SDS-PAGE sample buffer. The proteolytic products were analyzed by Western blotting.

### siRNA-mediated knockdown of calpain

Specific siRNAs targeting mouse calpain 1 and a non-targeted control siRNA were purchased from Santa Cruz Biotechnology (Santa Cruz, USA). Podocytes were seeded in a 6-well culture dish, and siRNA duplexes were diluted in 100 μl of serum-free medium. Then, 6 μl of Lipofectamine 2000 (Invitrogen, USA) was added to 100 μl of the siRNA duplexes. The duplexes were incubated for 15 min and subsequently added to each well. After 48 h of transfection, the cells were treated using the appropriate methods. After the cells were lysed in RIPA, Western blots were performed to examine protein levels.

### Statistical analysis

When two groups of data were compared, the independent samples t-test was applied. All data and images were analyzed using GraphPad Prism, version 5 (San Diego, CA, USA). A p-value less than or equal to 0.05 was considered significant, and all experiments were performed at least 3 times.

## Discussion

Calcineurin is a calcium-dependent phosphatase with a role in calcium signaling in diverse cells and organs, including podocytes and the kidney. Calcineurin is clinically important as a direct target of the immunosuppressive drugs CsA and tacrolimus [16]. CsA and tacrolimus have been widely used in the treatment of renal diseases due to their anti-proteinuric effects [1, 3]. However, the side effects of CsA and tacrolimus have led to further investigations on how to appropriately apply these drugs [28]. The important role of podocytes in proteinuria diseases suggests that the activation of local calcineurin in glomeruli and podocytes should be considered when utilizing these drugs. In this study, we examined calcineurin activity and expression during podocyte injury. The observed abnormal increase in calcineurin activity during podocyte injury was consistent with the beneficial effects of calcineurin inhibition for the treatment of proteinuria diseases. However, the protein expression of full-length 60-kDa calcineurin was notably down-regulated during podocyte injury. These contradictory changes suggest that there is another activated form of calcineurin that functions during podocyte injury.

The well-known activation of calcineurin depends on calcium and the binding of calmodulin. In the inactive state, the autoinhibitory domain occupies the catalytic site and blocks calcineurin activity. When calcium increases in the cytoplasm and calmodulin binds to the calcineurin binding sites, calcineurin undergoes a conformational change. The catalytic site is exposed, and the autoinhibitory domain is released [13]. For this type of activation, calcineurin must be intact. In addition to this traditional activation pathway, studies of nervous system and cardiovascular system diseases have reported another activation pathway that is independent of calmodulin binding. Both *in vivo* and *in vitro* studies have suggested that proteases, including calpain, can cleave and activate calcineurin in a proteolytic manner. This proteolytic effect on calcineurin removes the autoinhibitory domain from the full-length protein and transforms this phosphatase into a constitutively active form [17, 29, 30]. The contradictory changes in the activity and protein expression of full-length 60-kDa calcineurin suggest that protease-dependent activation may be involved in podocyte injury. To investigate this possibility, we examined the protease activity of calpain, and we found that calpain was abnormally activated during podocyte injury. In addition, Julie Peltier *et al*. showed that calpain activation promotes glomerular injury *in vivo* and increases proteinuria [19]. Taken together, these findings suggest that calpain is involved in podocyte injury. Calpain activation has been previously reported in studies of many different nervous system diseases. The pathogenesis of one of these diseases involves calcineurin cleavage and activation in the brain [29]. Both pharmacological inhibitors of calpain and specific siRNAs against calpain were used in the present study to explore whether calpain regulates calcineurin. As shown in Fig 3A–3C and 3G–3I, inhibition of calpain diminished the abnormal activity of calcineurin and restored the protein expression of full-length calcineurin. These results demonstrate that calpain regulates calcineurin during podocyte injury. In addition, inhibition of calpain also had an effect on recovering the injured F-actin (Fig 3F), which suggested blockade of calpain also has a therapeutic benefits on injured podocytes.

Our results suggest that a cleaved fragment of calcineurin was produced during podocyte injury due to cleavage by calpain. To confirm the presence of this fragment, purified human full-length calpain 1 protein was used to cleave proteins in podocytes injured by PAN, followed by detection of the corresponding protein band using Western blotting. Interestingly, a protein band was found above the location of the 42-kDa marker. Most importantly, when purified human calpain 1 was incubated with podocyte protein extract, the 60-kDa calcineurin band was decreased in a dose-dependent manner, and the 45-kDa protein fragment showed a compensatory increase. Previous studies of nervous system and cardiovascular system diseases have used mass spectrometry to show that the 45-kDa fragment is a product of calcineurin that is cleaved by calpain [17, 18]. In a study by Haiyan Wu *et al*., Western blotting and Coomassie Blue staining were used to identify 3 calcineurin fragments 57 kDa, 48 kDa, and 45 kDa in size after co-incubation of purified calcineurin and calpain at an appropriate temperature [17]. The detection of all of 3 fragments was confirmed by mass spectrometry. The 57-kDa calcineurin fragment was only detected after using a lower concentration of calpain. As the concentration of calpain increased, the 57-kDa fragment disappeared, and the levels of the 48-kDa and 45-kDa fragments were increased. In addition, the 45-kDa fragment was only detected in the absence of calmodulin, and both the 48-kDa and 45-kDa fragments were detected in the presence of calmodulin. In our study, the generation of the 45-kDa fragment was confirmed by the increase in this fragment at the expense of full-length calcineurin after calpain incubation and PAN treatment of podocytes. We did observe slight expression of the 57-kDa and 48-kDa proteins; however, we could not definitively confirm that these bands were cleavage products of calcineurin because whole protein including calcineurin, rather than purified calcineurin, was extracted from the podocytes. These results were similar to those of Hafiz et al., who evaluated the cleavage of calcineurin in whole human brain extracts but did not examine the cleavage of purified calcineurin [31]. Discrepant results may arise because cell and tissue extracts contain more complex proteases and endogenous inhibitors of calpain and calcineurin.

It is widely known that the activity of calpain is dependent on the level of calcium. They are distinguished by the calcium concentration required for in vitro activity: calpain 1 requires μm levels of calcium, while calpain 2 requires mM levels[32]. And also, according to Jun Oh et al elegant study, the level of calcium is important as stimulation of the calcium-sensing receptor stabilizes the podocyte cytoskeleton and improves cell survival[33]. In our study, though we have not accurately examined the effect of different levels of calcium on calcineurin, what we used siRNA and purified protein was all specific for calpain 1. As shown in Fig 3G and 3H, after silencing the calpain 1, the protein levels of 60 KDa calcineurin was recovered with treatment PAN. In addition, as shown in Fig 4A, the purified calpain 1 protein directly increased the cleavage calcineurin when incubated with podocytes extracts. Thus, we can conclude that calpain 1 can induce the calcineurin cleavage in podocyte injury.

Interestingly, as shown in Fig 4C–4F (lane 1), the 45-kDa calcineurin cleavage product was also detected in normal podocytes without PAN or ALLN treatment. This result suggests that calpain was also activated in normal unstimulated podocytes. Our data in Fig 3A and 3G lane 2 also confirm this finding, as we observed a slight increase in full-length 60-kDa calcineurin after inhibiting calpain with ALLN and calpain 1-specific siRNAs.

In summary, our study demonstrated that calcineurin activity is increased following PAN treatment of podocytes, while full-length 60-kDa calcineurin is down-regulated because over-activated calpain cleaves calcineurin into a 45-kDa fragment.
